# Identification of genes differentially expressed during interaction of resistant and susceptible apple cultivars (*Malus × domestica*) with *Erwinia amylovora*

**DOI:** 10.1186/1471-2229-10-1

**Published:** 2010-01-04

**Authors:** Angela Baldo, Jay L Norelli, Robert E Farrell, Carole L Bassett, Herb S Aldwinckle, Mickael Malnoy

**Affiliations:** 1 FEM-IASMA Research Centre, Via E. Mach 1, 38010 San Michele all'Adige (TN) Italy; 2 Department of Plant Pathology, Cornell University, 630 W. North St., Geneva, NY 14456 USA; 3 USDA-ARS Plant Genetic Resources Unit, 630 W. North St., Geneva, NY 14456 USA; 4 USDA-ARS Appalachian Fruit Research Station, 2217 Wiltshire Rd, Kearneysville, WV, 25430; 5 Pennsylvania State University, 1031 Edgecomb Avenue, York, PA, 17403 USA

## Abstract

**Background:**

The necrogenic enterobacterium, *Erwinia amylovora *is the causal agent of the fire blight (FB) disease in many Rosaceaespecies, including apple and pear. During the infection process, the bacteria induce an oxidative stress response with kinetics similar to those induced in an incompatible bacteria-plant interaction. No resistance mechanism to *E. amylovora *in host plants has yet been characterized, recent work has identified some molecular events which occur in resistant and/or susceptible host interaction with *E. amylovora*: In order to understand the mechanisms that characterize responses to FB, differentially expressed genes were identified by cDNA-AFLP analysis in resistant and susceptible apple genotypes after inoculation with *E. amylovora.*

**Results:**

cDNA were isolated from M.26 (susceptible) and G.41 (resistant) apple tissues collected 2 h and 48 h after challenge with a virulent *E. amylovora *strain or mock (buffer) inoculated. To identify differentially expressed transcripts, electrophoretic banding patterns were obtained from cDNAs. In the AFLP experiments, M.26 and G.41 showed different patterns of expression, including genes specifically induced, not induced, or repressed by *E. amylovora*. In total, 190 ESTs differentially expressed between M.26 and G.41 were identified using 42 pairs of AFLP primers. cDNA-AFLP analysis of global EST expression in a resistant and a susceptible apple genotype identified different major classes of genes. EST sequencing data showed that genes linked to resistance, encoding proteins involved in recognition, signaling, defense and apoptosis, were modulated by *E. amylovora *in its host plant. The expression time course of some of these ESTs selected via a bioinformatic analysis has been characterized.

**Conclusion:**

These data are being used to develop hypotheses of resistance or susceptibility mechanisms in *Malus *to *E. amylovora *and provide an initial categorization of genes possibly involved in recognition events, early signaling responses the subsequent development of resistance or susceptibility. These data also provided potential candidates for improving apple resistance to fire blight either by marker-assisted selection or genetic engineering.

## Background

Various defense responses are induced when a pathogen attempts to invade a non-host plant or resistant host. Among these induced responses the Hypersensitive Response (HR) is the most distinguishing hallmark of resistance and is characterized by rapid localized plant cell death at the site of infection [1,2]. The HR generates a physical barrier composed of dead cells and limits the availability of nutrients to the pathogen which can further restrict its spread. Other defense related responses often accompany HR, such as oxidative burst [3], the production of antimicrobial compounds (phytoalexins) [4], pathogenesis related proteins [5], and enzymes involved in the phenylpropanoid pathway [6].

The ability of some gram negative bacterial pathogens, such as *Erwinia*, *Pseudomonas, Xanthomonas *and *Ralstonia *strains, to cause disease in susceptible plants and elicit HR in resistant or non-host plants is governed by the *hrp*(hypersensitive reaction and pathogenicity) gene cluster [7,8]. These genes encode components of a type III secretion system involved in the secretion of effectors proteins [9]. These secretion pathways are used to deliver proteins from bacterial cytoplasm either to the culture media or into the host cell cytoplasm [10]. One of these bacteria, *Erwinia amylovora*), causes a bacteriosis, called fire blight, in species belonging to the subfamily *Maloideae *of the family *Rosaceae*, including apple (*Malus × domestica*), pear (*Pyrus communis *L.) and ornamentals such as cotoneaster and pyracantha. Fire blight has been known as a destructive disease of apple and pear for over 200 years [11]. Extensive information is available about the disease, including epidemiology, susceptibility of host genotypes [12] and in particular, the pathogen *E. amylovora *[13]. However, the biochemical and genetic basis leading to the disease or the establishment of resistance in the host plant are still relatively unknown. Indeed, as opposed to a number of other plant pathogen interactions, no specific R/avr gene-for-gene interactions have been described in relation to fire blight. This suggests that the resistance could be under polygenic control. Although no resistance mechanism to *E. amylovora *in host plants has yet been characterized, recent work has identified some molecular events which occur in resistant and/or susceptible host interaction with *E. amylovora*: i) massive oxidative stress is induced by *E. amylovora *with similar kinetics and magnitude as with an incompatible pathogen, regardless of the infected host genotype [14], and this elicitation requires both pathogenicity factors, *hrp*N and *dsp*A/E, of *E. amylovora *[15]; ii) some specific defense pathways, in particular specific branches of phenylpropanoid pathway leading to phytoalexin synthesis, are suppressed in the susceptible host by *E. amylovora*, whereas they are induced in the resistant host[16]; iii) *hrp*-independent defense responses that could be effective in stopping an infection of *E*. *amylovora *are delayed in susceptible hosts [17]; and iv) three pathogenesis-related (PR) genes of apple, PR-2, PR-5 and PR-8, are also induced in response to inoculation with *E. amylovora *[18]. Additionally, infection of apple by *E. amylovora *results in decreased photosynthetic efficiency. Forty-eight hours after inoculation with *E. amylovora *photosynthetic rates are reduced in both mature and young apple leaves measured under ambient CO_2_, whereas under saturating CO_2 _the photosynthetic rate is reduced only in young infected leaves; suggesting an inhibition of Photosystem (PS) II in both infected mature and young leaves and an inhibition of PS I only in infected young leaves [19]. Similarly, changes are observed in the chlorophyll fluorescence of *E. amylovora*-challenged apple leaves prior to the development of disease symptoms [20].

Earlier molecular investigations of the *E. amylovora- Malus *interaction have been limited to a restricted number of plant defenses previously characterized in other plant-pathogen interactions. To identify genes implicated in the control of fire blight resistance, we have chosen to use the RNA fingerprinting technique of cDNA amplified fragment length polymorphism (cDNA-AFLP) [21]. This technique was applied to study the genes differentially regulated in susceptible 'M.26' (compatible) and resistant Geneva 'G.41' (incompatible) apple rootstocks [22] following challenge with a virulent strain of *E. amylovora *(Ea273) or buffer. Gene expression was studied 2 and 48 hours after inoculation of the leaves by wounding. The purpose of this study was to understand the mechanisms of interaction between *Malus *and *E. amylovora *in resistant and susceptible apple cultivars. The results will aid in the design of new strategies to improve apple resistance to *E. amylovora*, and facilitate development of molecular tools for marker-assisted selection.

## Results

To elucidate the molecular and biochemical mechanisms involved in resistance and susceptibility of apple trees to *E. amylovora*, a comparison of gene expression patterns between the resistant apple rootstock 'G.41' and the susceptible 'M.26' was carried out using cDNA-AFLP-analysis at 2 and 48 hpi. These time points were selected based upon previous analysis of the temporal transcriptional response of *Malus *to *E. amylovora *[23]which indicated that basal defense to pathogen associated molecular patterns (PAMPs) occurred within 1-2 hpi whereas expression of PR proteins occurred 24-48 hpi.

**Figure 1:**
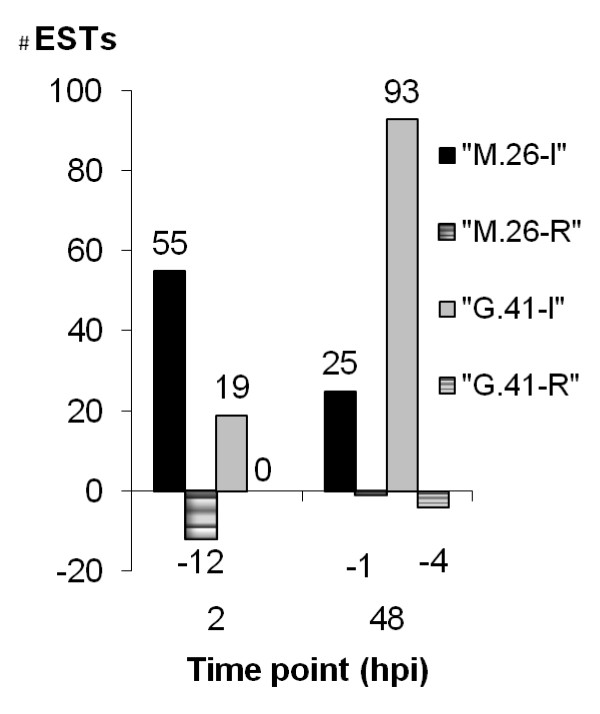
**Distribution of cDNA-AFLP fragments up (induced, I) and down (repressed, R) regulated in fire blight susceptible "cv. M.26" and resistant "cv. G41" apple rootstocks**. Down regulated fragments are designated by a minus sing (-); no down regulated cDNA sequences were identified in "cv. G41", and hpi = hours post inoculation.

cDNA templates were prepared from leaves inoculated with *E. amylovora*, and from control leaves treated with buffer for both apple cultivars. A total of 42 different primer combinations of *Mse *I primers having 2 selective nucleotides at their 3'-ends were applied. This resulted in the capture of approximately one thousand cDNA fragments, ranging in size from 40 to 1200 bp. Each cDNA fragment generated an average of 30 discrete and clearly visible bands when amplified with a given AFLP primer combination. Overall, cDNAs isolated from the "M.26" and "cv. G.41"apple cultivars displayed almost identical patterns on the polyacrylamide gel with a given primer combination in at least two independent experiments. However, a comparison of cDNA-AFLP patterns revealed the following differences: i) of the approximately one thousand cDNA fragments detected on cDNA-AFLP gels, 205 bands were differentially up- or down-regulated between the two cultivars, ii) fifty-five fragments were up regulated 2 hpi in the susceptible cultivar "cv. M.26", whereas only 19 were up-regulated in the resistant cultivar "cv. G.41" at the same time and iii) at 48 hpi more fragments were up- regulated in "cv. G.41" (93 fragments) compared to "cv. M.26" (25 fragments) and only one down-regulated fragment were observed in "cv. M.26" (Fig. 1). Most of all the down-regulated fragments were found in the susceptible cultivar "cv. M.26"and most were found 2 hpi (12). These bands were excised from the silver-stained gel, re-amplified, and cloned into a plasmid vector.

The differentially expressed cDNA sequences were assigned to broad functional categories based on similarity comparison to the Genbank Non-Redundant protein database using BLASTx. Table 1 shows the classification of the differentially expressed genes identified from both "cv. M.26" and "cv. G.41". For the largest group of clones (41%) no functional motifs or homologues were identified in the database. The next most abundant group (15%) were clones with similarity to genes involved in photosynthesis, followed by two groups of genes (12% each) involved in general metabolism and having similarity to genes associated with plant stress responses. Finally, a number of clones were identified with similarity to genes involved in signaling pathways (5%), energy (4%), protein metabolism (4%) and transport (1%). The distribution of genes in the various categories may be biased by the relative numbers of annotated genes in the database for each category. However, it is clear that over half of the genes identified in this study could be placed into a potential functional category based on similarity to previously characterized genes.

**Table 1:** Broad functional classification of the differentially expressed genes identified in "cv. M.26" and "cv. G.41".

Functional class	% of total
Unknown and unclassified	41
Photosynthesis	15
General metabolism	12
Defense	12
Signaling	6
Nucleic metabolism	5
Energy	4
Protein metabolism	4
Transport	1

The positive BLASTx hit results for the differentially expressed genes are shown in additional file 1 for "cv. M.26"and "cv. G.41". Sequences with no significant similarity to known genes are not included. A number of the cDNA-AFLP fragments identified with different primer sets were subsequently found to be identical sequences. ESTs found in both genotypes were not included in additional file 1, such as ferredoxin, cytochrome b6 and ribulose 1,5-bisphosphate. BLASTx matches with high e-values were obtained for 83 unique sequences that were differentially expressed between the two genotypes, making it difficult to determine which of these ESTs are specifically involved in the resistance or susceptibility to fire blight. To narrow this list we used a candidate gene approach, in which the contigs from fire blight challenged tissue were compared against the ESTs from unchallenged tissue and the resulting BLASTn scores were ranked from lowest to highest. The expectation is that some of the sequences which do not match contigs from healthy tissue are expressed preferentially under disease conditions (Table 2, column A). Sequences from fire blight-challenged tissue with the top 16 lowest match scores to sequences from healthy tissue were identified as potential candidates (BLASTn score below 100). As described by Norelli et al. 2009, several other datasets were compared using BLASTn to annotate the contigs from infected tissue: i) genes associated with avirulent *Pseudomonas syringae *infection of *Arabidopsis *(Table 2, column C), ii) genes associated with virulent *P. syringae *infection of *Arabidopsis *(Table 2, column B), iii) genes associated with the salicylic acid response in *Arabidopsis *(Table 2, column D), and iv) ESTs derived from the suppression subtractive hybridization (SSH) disease-time course experiments (Table 2, column E) discussed below. In addition, a single sequence was selected from each NCBI apple Unigene set that contained ESTs isolated for *E. amylovora *infected tissue and had an NCBI annotation associated with a known disease resistance pathway. Each of these sequences was also compared against the contigs from infected tissue using BLASTn (data not shown) [These comparisons suggested that the ESTs may be specifically involved in the interaction between *Malus *and *E. amylovora*, i.e. in basal defense response, or in the compatible or incompatible interaction, i.e. resistance (Table 2)]. A threshold superior to 100 of the BLASTN score (Table 2) was used to consider that an EST was expressed in response to one of the condition previously described (red box in table 2).

**Table 2:** Similarity of cDNA-AFLP sequences to a variety of datasets:

Fragment ID	Gene annotation	Dataset Comparison				
		A	B	C	D	E
		BLASTn	BLASTx	BLASTx	BLASTx	BLASTn
176.2-G41-48I	putative disease resistance protein [Malus × domestica]	**34**	25	22	21	28
176.1-G41-48I	unknown [Malus × domestica]	**34**	24	23	23	30
171-G41-48I	Probable WRKY transcription factor 53 (WRKY DNA-binding protein 53)	**36**	24	18	24	30
54.2-M.26R	DNA topoisomerase II [Malus × domestica]	**36**	24	20	24	26
175-G41-48I	putative WRKY transcription factor 30 [Vitis aestivalis]	**38**	26	23	24	32
131.4_G41_48_OE	hypothetical protein pNG7269 [Haloarcula marismortui ATCC 43049] gb|AAV44969	**38**	20	20	23	28
37-G41-48R		**40**	20	18	22	28
136.2-G41-2I	hypothetical protein 12.t00009 [Asparagus officinalis]	**40**	24	21	23	26
64.4-G41-48OE	Fusarium resistance protein I2C-5-like [Oryza sativa (japonica cultivar-group)]	**42**	24	21	22	28
201.3-G41-48I	putative leucine-rich repeat transmembrane protein kinase [Malus × domestica]	**44**	41	39	21	30
200.1-G41-48I	Probable WRKY transcription factor 29	**52**	64	22	54	26
213-G41-48I	Probable WRKY transcription factor 65 (WRKY DNA-binding protein 65)	**56**	68	22	52	26
221-G41-48I	Probable WRKY transcription factor 65 (WRKY DNA-binding protein 65)	**56**	66	22	51	30
7.2_M.26_2	hypothetical protein RT0201 [Rickettsia typhi str. Wilmington] gb|AAU03684.1| cons	**64**	21	21	22	62
190-G41-48I	Leucine-rich repeat [Medicago truncatula]	418	21	22	22	28
175.2_G41_48I	beclin 1 protein [Malus × domestica]	541	22	23	30	30
81_G41_48I	AT5 g56010/MDA7_5 [Arabidopsis thaliana]	841	22	23	30	**769**
176.3_G41_48I	protein kinase [Malus × domestica]	280	22	25	30	26
171.1_G41_48I	protein kinase [Malus × domestica]	107	23	24	35	28
4.3_M.26_2I	MYB11 [Malus × domestica]	628	24	21	24	**646**
165_M.26_2R	protein kinase [Malus × domestica]	168	24	44	51	28
201_M.26R	LYTB-like protein [Malus × domestica]	692	24	24	24	26
98_G41_48	putative chalcone isomerase 4 [Glycine max]	1195	24	22	26	**805**
3.3_M.26_2I	Os08 g0162600 [Oryza sativa (japonica cultivar-group)]	289	24	22	20	**289**
137.1_G41_48_I	dbj|BAC57824.1| unknown	472	25	25	27	24
115_G41_2I	chalcone synthase [Malus × domestica]	714	26	24	22	26
200_G41_48I	soluble NSF attachment protein [Malus × domestica]	496	26	25	26	28
4.2_M.26_2I	ATP binding/kinase/protein serine/threonine kinase [Arabidopsis thaliana	936	29	26	29	**936**
142_G41_48I	flag-tagged protein kinase domain of putative mitogen-activated protein kinase kinase	654	37	22	34	**293**
166_M.26_2R	protein kinase [Malus × domestica	414	44	49	20	26
1.2_M.26_2I	putative hydroquinone glucosyltransferase; arbutin synthase [Malus × domestica]	444	62	23	60	32
112_G4148I	aquaporin 2 [Bruguiera gymnorhiza]	793	**116**	**166**	21	34
201_G41_48I	translation initiation factor eIF-4A [Malus × domestica]	309	**121**	24	21	30
137.2_G41_48I	hypothetical protein [Citrus × paradisi]	507	**141**	23	21	**498**
205_G41_48I	glyceraldehyde-3-phosphate dehydrogenase [Panax ginseng]	765	**176**	22	24	**414**

ESTs expressed preferentially under fire blight challenge (A), *A. thaliana *compatibility ESTs (B); *A. thaliana *incompatibility ESTs (C), similar to *A. thaliana *Salicylic Acid Response ESTs (D), and *Malus *EST in tissue challenged by *E. amylovora *found by Norelli *et al*, (2009) by suppression subtractive hybridization (SSH) (E). Gene annotations were determined by most informative BLASTx comparision below a predetermined threshold of 1*e*^-3^. NA indicates BLASTn similarity score below (A) or above (B-E).

**Figure 2:**
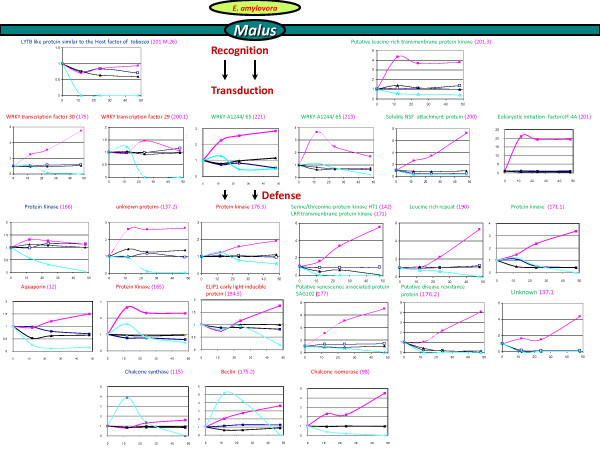
**Time course of cDNA-AFLP fragment abundance during the *E. amylovora - Malus *host-pathogen interaction**. The possible involvement of specific genes in resistance or susceptibility mechanisms was inferred from their response in fire blight resistant "cv. G41" (■ symbol) and susceptible "cv. M.26" (Δ symbol) (see Discussion). Black lines indicated response in mock-inoculated leaf tissue, whereas red and blue lines *E. amylovora*-inoculated "cv. G41" and "cv. M.26", respectively. X-axis represents hours post inoculation (hpi) and y-axis relative gene expression (see Materials and Methods). Numbers in brackets following gene annotation refer the fragment ID number in additional file 1.

Twenty eight genes candidate resistance/susceptibility genes were selected and their expression profiles by qRT-PCR (Figure 2). Quantitative RT-PCR analysis of the same cDNAs used for AFLP analysis (2 and 48 hpi) confirmed the profile of expression observed by AFLP for 79% of the 28 ESTs analyzed (Table 3). Additionally, cDNAs isolated from the same biological experiment at 12 and 24 hpi were included for a time course analysis (Fig. 2). Looking at the putative function of the 32 genes tested by qPCR and their pattern of expression, we suggested in the figure 2 a possible representation of involvement of these genes dureint the interaction *Malus E. amylovora*. It is possible to identified 3 classes of genes expressions, i) genes repress or activated only in the susceptible cultivars, M.26 (labeled in blue, Figure 2), ii) genes only activated in the resistant cultivars G.41 (labeled in green, Figure 2) and genes activated in G.41 and repress in M.26 (labeled in red, Figure 2). It's interesting to observed form the pattern of expression of these genes that most the genes induced in the resistant cultivars G.41 are expressed 24 h post inoculation [such as the EST soluble NSF attachment protein (200), leucine rich protein (190), Serine/threonine-protein kinase HT1 (142) or the Protein kinase (171.1)]. Few are induced early such as WRKY-A1244/65 (213), Putative leucine-rich transmenbrane LYTB like protein similar to the Host factor of *tobacco *(201 M.26) and the protein kinase (201.3). In opposite most of the genes repress in the susceptible cultivars seems to be down regulated after or before 12 h post inoculation [such as the Putative leucine-rich transmenbrane LYTB like protein similar to the Host factor of *tobacco *(201 M.26), or the protein kinase (201.3)].

**Table 3:** Genes found differentially expressed by AFLP confirmed by qRT-PCR

cDNA sequence and annotation	AFLP profile		Confirmed by qRT PCR
	cv. M.26	cv. G.41	
165-M.26-2R protein kinase	2 R		N
166-M.26-2R protein kinase	2 R		Y
175-G41-48I putative WRKY transcription factor 30		48 I	Y
200.1-G41-48I Probable WRKY transcription factor 29		48 I	Y
213-G41-48I Probable WRKY transcription factor 65		48 I	Y
221-G41-48I WRKY-A1244		48 I	Y
200-G41-48I Soluble NSF attachment protein		48 I	Y
142-G41-48I Serine/threonine-protein kinase HT1		48 I	Y
171.1-G41-48I protein kinase		48 I	Y
137.2-G41-48I hypothetical protein B2		48 I	Y
176.3-G41-48I protein kinase		48 I	Y
171-G41-48I putative leucine-rich repeat transmembrane protein kinase		48 I	Y
201.3 G.41-48I Putative leucine-rich repeat transmembrane protein kinase		48 I	Y
190-G41-48I Leucine-rich repeat		48 I	Y
176.2-G41-48I putative disease resistance protein		48 I	Y
201-G41-48I translation initiation factor eIF-4A		48 I	Y
201-M.26R LYTB-like protein	48 R		Y
12-G41-48I putative aquaporin		48 I	Y
175.2-G41-48I beclin 1 protein		48 I	Y
177-G41-48I putative senescence-associated protein SAG102		48 I	Y
4.3-M.26-2-I MYB11	2 I		N
194.5-G41-48I ELIP1 (early light inducible protein)		48 I	Y
98-G41-48I chalcone isomerase 4		48 I	Y
115-G41-2I chalcone synthase		2 I	Y
55.2-M.26R SIR2-family protein	R		N
137.1-G41-48I unknown		48 I	N
176.1-G41-48I unknown		48 I	N
84.2-M.26-2I unknown protein	2 I		N

## Discussion

Understanding the complex transcriptional changes occurring in *Malus *in response to *E. amylovora *is important for efficient management of this pathogen. In this study, we used cDNA-AFLP to identify genes up- or down-regulated in resistant and susceptible apple cultivars after inoculation with *E. amylovora. *cDNA-AFLPs have advantages over other commonly used gene display methods (for a review see [24]). This technique can be performed in the absence of DNA sequence data and, as a PCR based method, only requires minute amounts of RNA. It also allows direct comparison between distinct genotypes, which is often difficult by subtractive cDNA techniques. Because of the use of stringent annealing conditions during PCR, cDNA-AFLP banding patterns are highly reproducible compared with, for example, differential display PCR [25]. This technique has been used with success in apple to study the rootstock effect on gene expression patterns in apple tree scions [26], the interaction between rosy apple aphids and *Malus *[27], and to find an apple gene that contributes to lowering the acidity of fruit [28].

Using a total of 42 different primer combinations, 198 different cDNA-AFLP fragments were identified between the resistant ('G.41') and susceptible ('M.26') apple cultivars after inoculation with *E. amylovora*. Among the genes selected for verification by qRT-PCR, the pattern of expression was nearly identical in mock inoculated 'G.41' and 'M.26', suggesting that differentially expressed cDNA-AFLP fragments were not due to genetic differences between the two cultivars. If the 2,800 genes regulated in response to bacterial pathogen inoculation in the *A*. *thaliana*-*Pst *DC3000 host pathogen system [29]are used as an estimate for the number of genes expected to respond in the *Malus-E. amylovora *interaction, this study identified approximately 7% of the genes regulated in response to pathogen challenge. The relatively low level of transcriptome coverage in this study was probably due to the limited number of time points analyzed (2 and 48 hpi), as well as the specific time points selected for analysis. In *A. thaliana *the greatest gene expression in response to *Pst *DC3000 occurs 12 hpi and involves approximately 2700 genes over all time points [30,31]. Additionally, the labor- intensive nature of cDNA-AFLP analysis and the finite number of primer pairs that can feasibly be used limits the number of ESTs that can be detected. With the development of an apple genome sequence [32], short-read, high-throughput sequencing technologies such as (*RNa-seq 454 *technology) should allow greater coverage of the apple transcriptome following *E. amylovora *infection in future studies.

cDNA-AFLP analysis results in EST sequences that do not represent the entire gene transcript. Using the Malus unigene most similar to the shorter EST for blastx comparisons was useful in improving the reliability of BLAST analysis and expanding the amount of biological information derived from the cDNA-AFLP ESTs. In general, using the Malus unigene most similar to the EST for blastx comparisons was most informative when the EST contained primarily 3'-untranslated region sequence. When cDNA sequence was available, blastn comparisons to the NCBI nr database usually produced equivalent results to blastx comparisons using the Malus unigene most similar to the EST. However, for species which lack extensive cDNA and genomic sequence data, such as apple, the utility of blastn comparisons is limited. Despite the utility of using the Malus unigene most similar to the EST for blastx comparisons, caution is needed in interpreting these BLAST results [23].

This study has provided a preview of the genes associated with the interaction between *Malus *and *E. amylovora*. The cDNA-AFLP sequences identified were assigned to broad functional categories based on database similarity (Table 1 and additional file 1). The percentage of each category is similar to what has been reported for the interaction between *Malus *and *Pseudomonas fluorescens Bk3 *[33], and is also consistent with previous studies on the interaction between *Malus *and *E. amylovora *[16,23,34]. In agreement with the work of Venisse *et al *[16], we observed that genes involved in the phenylpropanoid pathways were up-regulated in the resistant cultivars in response to *E. amylovora*. Also, some of the defense-related and signaling genes, such as protein kinase, soluble NSF attachment protein, putative leucine rich repeat transmembrane protein kinase, and the putative disease resistance protein, aquaporin, were also found to be up- or down- regulated in a similar study comparing the response of the resistant apple cultivar 'Evereste' to the susceptible rootstock 'MM.106' [14]. However, in contrast to the work of Venisse *et al *[16] and Bonasera *et al *[18], no PR genes were found up-regulated in the susceptible or resistant cultivars. This can be attributed to the fact that we did not use all the possible AFLP primer combinations or that the genes were similarly regulated at the time points analyzed in this study.

Fifteen percent of the cDNA-AFLP sequences identified in this study were involved in photosynthesis. The induction of some photosynthetic genes during the interaction between resistant *Malus *and *E. amylovora *may implicate light-sensing mechanisms in the induction of plant disease defense signaling. Current models of mechanisms of plant defense against pathogen infection are based on animal models, and rarely consider light signaling pathways or photo-produced H_2_O_2 _and other reactive oxygen species (ROS) [35]. Plant defense against pathogen infection has been shown to be linked to the light-sensing network and to the oxygen-evolving complex in Photosystem II (PSII) [36,37], and PSII plays an important role in preventing the accumulation of ROS [38]. Frequently ROS are needed to trigger protective responses, such as the down-regulation of PSII activity [39,40] and to induce systemic acquired resistance. During an incompatible interaction, the burst of ROS can trigger an array of defense responses including a hypersensitive reaction. In the case of the compatible interaction between *E. amylovora *and a host plant (pear or apple), bursts of ROS seem to be paradoxically necessary for a successful colonization of the plant by this bacterium [34]. This burst is the result of the combined action of two hrp effectors of *E. amylovora *HrpN_Ea _and DspA/E [15]. An increase in photosynthetic activity stimulates the production of ATP and sugar. This suggests that *Malus × domestica *may prevent the colonization by *E. amylovora *by increasing host plant defense via the light sensing signaling pathway and by activation of additional defense related genes. In the case of interaction with fire blight, the transcriptional up-regulation of photosynthesisrelated genes is similar to that observed during the interaction between *Arabidopsis thaliana and Pseudomonas syringae *[29,31].

To identify potential candidate genes involved in host resistance mechanisms against *E. amylovora *we conducted a bioinformatics analysis to compare the cDNA-AFLP ESTs with all the non-fire blight associated ESTs at NCBI, with the ESTs found previously during the *Malus -E.amylovora *interaction, with SSH ESTs activated in *A. thaliana *during a compatible interaction, with SSH ESTs activated in *At *during an incompatible interaction, with SSH ESTs activated in *A. thaliana *during SAR, and with ESTs previously identified during the interaction between *Malus *and *E. amylovora *(Table 2). This approach allowed us to determine that 90 of the cDNA-AFLP ESTs were specifically involved in the interaction between *Malus *and *E. amylovora*, either in basal defense response or in compatible or incompatible interaction. Most of these ESTs were not identified in a similar SSH analysis [23]. This indicates that these two techniques are complementary, but could also be due to the partial transcriptome coverage reported in both this cDNA-AFLP and the SSH study [23].

Of the 90 cDNA-AFLP sequences identified by bioinformatics, 32 were selected for confirmation by qRT-PCR. The different genes were assigned in different mechanism according what was reported in the literature. This analysis confirmed the expression profile predicted by AFLP for the ESTs analyzed and identified three classes of expression profiles. The first, and perhaps most interesting class of ESTs was only activated in the resistant cultivar, such as 176.2-G41-48I (putative disease resistance protein [*Malus × domestica*]) and 137.1-G41-48I (similar to Os08 g0162600 Rubredoxin-type Fe(Cys)4 protein family protein [*Oryza sativa *(*japonica *cultivar-group)]) (Fig. 2). These genes are good resistant gene candidates for fire blight. The second class contained ESTs activated at different times in the resistant cultivar than in the susceptible cultivar and repressed in the susceptible cultivar between 12 and 48 hpi depending on the ESTs, such as 200.1-G41-48I (probable WRKY transcription factor 29) and 137.2-G41I-48I (hypothetical protein [*Citrus *× paradise]) (Fig. 2). These genes could be involved in the response of the plant that contributes to the rate of symptom development and possible resistance. The third class contained ESTs that were only repressed in the susceptible rootstock M.26, such as 55.2-M.26R- (SIR2-family protein [*Malus × domestica*]) (data not shown). The pattern of expression of 2 of these genes [(Chalcone syntahse (115), and Chalcone isomerase (98)] confirms the results of Venisse et al. (2002). These genes could possibly be useful as susceptibility markers. The profile of expression of other ESTs will be verified in the future.

## Conclusion

The overall goal of this project was to characterize the genomic response of apple to fire blight. These data are being used to develop hypotheses of resistance or susceptibility mechanisms in *Malus *to *E. amylovora *and provide an initial categorization of genes possibly involved in recognition events, early signaling responses the subsequent development of resistance or susceptibility (Fig. 2). Further analysis of these genes will help us understand the complex mechanisms of resistance or susceptibility that apple activates during infection by *E. amylovora. *The data also provide potential candidates for improving apple resistance to fire blight either by marker-assisted selection or genetic engineering. Future studies will determine if these genes co-localize with resistance loci or QTLs and how strategies might be developed to incorporate these genes into breeding programs.

## Methods

### Plant material

The two rootstock "cv. M.26" and "cv. G.41" (G3041) were chose for their different level of susceptibility to *Erwina amylovora*[41]. One-year-old potted apple trees of " cv. M.26" EMLA and " cv. G.41" rootstock were grown in a growth chamber as described by Norelli et al. 2009, except that prior to treatment trees were visually evaluated for growth vigor and divided into equal vigor blocks of 5 replicate trees for each cultivar-challenge treatment-sample time (total of 20 blocks).

### Challenge treatments and sampling

*E. amylovora *and buffer challenge treatments were applied by transversely bisecting leaves as described by Norelli et al. [23]. Leaf tissue samples were collected 2 hours post inoculation (HPI), 12 hpi, 24 hpi and 48 hpi. Temporal synchrony of sample tissue was achieved by limiting the sample tissue to a 3-6 mm wide strip of leaf tissue cut parallel to the original inoculation cut, as described by Norelli et al. [23].

### RNA isolation

Leaf samples were pooled prior to RNA isolation, and RNA was isolated from challenged leaf tissue using the Concert Plant RNA Reagent (Invitrogen #451002) as described by Norelli et al. 2009. Double stranded cDNAs were constructed using SuperSMART cDNA Synthesis Kit (BD Bioscience Clontech#K1054-1) as described by Bassett et al. [42].

### AFLP analysis

cDNA-AFLP experiments were conducted using the Licor procedure (Li-Cor, ALFP IRDey 800 #830-06194). Double stranded cDNA was digested with *Mse *I and *EcoRI *restriction endonucleases, followed by the addition of an adaptor. The specific PCR amplification was done with 2 to 3 selective base primers present in the kit. Amplification products were separated on a 6% polyacrylamide gel run at 80 W until the bromophenol blue reached the bottom of the gel and then visually displayed by silver staining. Polymorphic bands were excised from the dried gel and re-amplified following the same PCR conditions and primer combinations. The amplified DNA fragments were examined by agarose gel electrophoresis, cloned into pGEM-easy T vector (Promega, USA) and sequenced.

### Candidate gene identification

The entire set of *Malus *ESTs was downloaded from NCBI, screened for vector and organelle contamination according to Norelli, et. al [23] and separated according to whether the tissue of origin was reported to be challenged with fire blight, or not. The resulting two subsets of ESTs were compared using BLASTn. Sequences of genes associated with *Arabidopsis *disease response (*P. syringae *challenge and salicylic acid response) were downloaded from the Arabidopsis Information Resource [43] according to Norelli et. al [23].

### Confirming the pattern of expression of differentially expressed cDNA-AFLP ESTs

Quantitative reverse transcriptase PCR (qRT-PCR) analyses were performed with an IQTM5 Real Time PCR detection system (BIO-RAD, Hercules, CA) in a 25 μl volume containing 3 μl of cDNA, and 22 μl of the PCR master mixture. The PCR master-mixture contained the following: 0.5 μM of each reverse and forward primers, 0.2 mM dNTPs, 5 mM MgCl_2_, 2× SYBR Green I (Molecular Probes:http://www.probes.com) for the quantification of the gene expression, 2.5 μl hot start *Taq *polymerase buffer (10×), and 0.2 μl Takara Ex Taq Hot start Version (Takara, Madison, WI). PCR conditions for amplifying gene candidate DNA were 95°C for 1 min, then 50 cycles of 95°C for 10s, and 60°C for 60 sec, and for EF gene (used as an endogenous control) were 95°C for 1 min, then 50 cycles of 95°C for 10 sec, 54°C for 60 s. The primer pairs for each gene analyzed are provided in supplementary material (additional file 2). Sequences generated were deposited in GenBank [44] (Accession Nos. EX978970-EX9820069 additional file 1).

The specific amplification was evaluated by melt curve analysis and agarose gel electrophoresis. No primer dimmers were obtained, and only one product was amplified from each analyzed gene. To determine the amplification efficiencies and correlation efficiencies of each PCR reaction, a serial dilution series of cDNA of all samples was analyzed. The efficiencies and the calculation of the expression level were estimated using the iQ5 Optical System Software 2.0 (Bio-Rad) according to Vandesompele et al. [45]. For rime point the transcription level was quantified relatively using the primers mentioned in additional file 2. All samples were normalized using Elongation factor EF1α mRNA as internal control samples for each gene. The scaling of the gene expression for each sample was performed relative to the mRNA expression level at the time 0 h for each treatment. Relative gene expression was expressed as fold change in comparison to mock challenged M.26 at 2 hpi [46].

## Authors' contributions

AB carried out all the bio-informatics analysis and participated in writing the first manuscript draft, and its revision. JLN participated in the experimental design, carried out the plant inoculation and RNA extraction, and contributed to writing of the manuscript and its revision. REF carried out the cDNA synthesis, and contributed to the manuscript revision. CB and HSA participated in the experimental design, and contributed to the manuscript revision. MM. Conceived the study, participated in the experimental design, carried out molecular biology work, participated in the coordination of the work, helped to draft the manuscript and contributed to its revision. All authors read and approved the final manuscript

## Supplementary Material

Additional file 1 **Bioinformatic annotation of cDNA-AFLP ESTs identified as differentially regulated in the *Malus *- *E. amylovora *host-pathogen interaction**. list of clones differentially expressed during the interaction *Malus Erwinia amylovora *obtained by cDNA-AFLP, In this table is reported the size of each clones cloned, the NCBI accession number of each sequences, the pattern of expression, the Blast annotation of each sequence and their e values. Click here for file

Additional file 2 **DNA sequence of forward and reverse PCR primers used to confirm differential expression of specific ESTs**. list of primer developed to study the expression of each specific EST which seems to be specifically activated or repressed during the interaction *Malus Erwinia amylovora.* Click here for file
