# Supplementary material for: Identification of genes differentially expressed during interaction of resistant and susceptible apple cultivars (Malus × domestica) with Erwinia amylovora
Source: BMC Plant Biol. 2010 Jan 4;10:1. doi: 10.1186/1471-2229-10-1 (PMC2827420; doi:10.1186/1471-2229-10-1)
Supplement: Additional file 1 — Bioinformatic annotation of cDNA-AFLP ESTs identified as differentially regulated in the Malus - E. amylovora host-pathogen interaction. list of clones differentially expressed during the interaction Malus Erwinia amylovora obtained by cDNA-AFLP, In this table is reported the size of each clones cloned, the NCBI accession number of each sequences, the pattern of expression, the Blast annotation of each sequence and their e values. [file 1471-2229-10-1-S1.DOC]

Table S1: Bioinformatic annotation of cDNA-AFLP ESTs identified as differentially regulated in the *Malus* - *E. amylovora* host-pathogen interaction.

| Clone | GEnBank Accn | Size bp | Genotype | | | | expression | BLAST annotation | E value |
| --- | --- | --- | --- | --- | --- | --- | --- | --- | --- |
| **Signaling** |  |  |  | | | |  |  |  |
| 165-M.26-2R | EX978970 | 370 | M.26 | | | | 2h R | Protein kinase [Malus x domestica] | 2.00E-023 |
| 166-M.26-2R | EX978971 | 229 | M.26 | | | | 2h R | Protein kinase [Malus x domestica] | 2.00E-023 |
| 175-G41-48I | EX978972 | 342 | G.41 | | | | 48h I | Putative WRKY transcription factor 30 [Vitis aestivalis] | 2.00E-023 |
| 200.1-G41-48I | EX978973 | 210 | G.41 | | | | 48h I | Probable WRKY transcription factor 29 (WRKY DNA-binding protein 29) | 1.00E-027 |
| 213-G41-48I | EX978974 | 570 | G.41 | | | | 48h I | WRKY-A1244 [Capsicum annuum] | 4.00E-042 |
| 221-G41-48I | EX978975 | 500 | G.41 | | | | 48h I | WRKY-A1244 [Capsicum annuum] | 2.00E-041 |
| 200-G41-48I | EX978976 | 270 | G.41 | | | | 48h I | Soluble NSF attachment protein [Malus x domestica] | 7.00E-047 |
| **Defense related genes** | |  |  | | | |  |  |  |
| 142-G41-48I | EX982119 | 409 | G.41 | | | | 48h I | Serine/threonine-protein kinase HT1 (High leaf temperature protein 1) | 9.00E-033 |
| 171.1-G41-48I | EX982120 | 477 | G.41 | | | | 48h I | Protein kinase [Malus x domestica] | 2.00E-023 |
| 176.3-G41-48I | EX982071 | 240 | G.41 | | | | 48h I | Protein kinase [Malus x domestica] | 2.00E-023 |
| 171-G41-48I | EX982072 | 192 | G.41 | | | | 48h I | Putative leucine-rich repeat transmembrane protein kinase [Malus x domestica] | 2.00E-023 |
| 201.3-G41-48I | EX982073 | 182 | G.41 | | | | 48h I | Putative leucine-rich repeat transmembrane protein kinase [Malus x domestica] | 2.00E-028 |
| 190-G41-48I | EX982074 | 523 | G.41 | | | | 48h I | Leucine-rich repeat [Medicago truncatula] | 4.00E-034 |
| 176.2-G41-48I | EX982075 | 240 | G.41 | | | | 48h I | Putative disease resistance protein [Malus x domestica] | 2.00E-023 |
| 201-G41-48I | EX982076 | 160 | G.41 | | | | 48h I | Translation initiation factor eIF-4A [Malus x domestica] | 1.00E-022 |
| 201-M.26R | EX982050 | 369 | M.26 | | 2 and 48h R | | | LYTB-like protein [Malus x domestica] | 6.00E-031 |
| 4.2-M.26-2I | EX982051 | 519 | M.26 | | | | 2h I | ATP binding / kinase/ protein serine/threonine kinase [Arabidopsis thaliana] | 7.00E-060 |
| 81-G41-48I | EX982084 | 460 | G.41 | | | | 48h I | Heat shock protein 81-3 (HSP81-3) (HSP81.2) | 8.00E-078 |
| 112-G41-48I | EX982085 | 400 | G.41 | | | | 48h I | Aquaporin 2 [Bruguiera gymnorhiza] | 3.00E-061 |
| 12-G41-48I | EX982086 | 803 | G.41 | | | | 48h I | Putative aquaporin [Vitis vinifera] gb|AAW02943.2| aquaporin [Vitis vinifera] | 1.00E-114 |
| 175.2-G41-48I | EX982087 | 282 | G.41 | | | | 48h I | Beclin 1 protein [Malus x domestica] | 2.00E-023 |
| 74.1-G41-48I | EX982088 | 140 | G.41 | | | | 48h I | Nucleoporin [Lotus japonicus] | 0.005 |
| 172-G41-48I | EX982125 | 460 | G.41 | | | | 48h I | Superoxide dismutase [Mn] | 2.00E-023 |
| 91-G41-48I | EX982089 | 251 | G.41 | | | | 48h I | Cysteine proteinase [Populus tomentosa] | 7.00E-041 |
| 177-G41-48I | EX982090 | 342 | G.41 | | | | 48h I | putative senescence-associated protein SAG102 [Malus x domestica] | 2.00E-023 |
| 137.2-G41-48I | EX982112 | 302 | G.41 | | | | 48h I | Hypothetical protein [Citrus x paradisi] | 2.00E-038 |
| **Stress related protein** | |  |  | | | |  |  |  |
| 152-M26-2I | EX982055 | 329 | M.26 | | | | 2h I | Metallothionein-like protein [Pyrus pyrifolia] | 5.00E-018 |
| 137.1-G41-48I | EX982109 | 294 | G.41 | | | | 48h I | Rubredoxin-type Fe(Cys)4 protein family | 3.00E-025 |
| **Photosynthesis** |  |  |  | | | |  |  |  |
| 194.5-G41-48I | EX982091 | 496 | G.41 | | | | 48h I | ELIP1 (EARLY LIGHT-INDUCABLE PROTEIN); chlorophyll binding [Arabidopsis thaliana] | 4.00E-045 |
| 205.1-G41-48I | EX982092 | 232 | G.41 | | | | 48h I | PSI P700 apoprotein A2 [Lycopersicon esculentum] | 2.00E-005 |
| 89-G41-48I | EX982093 | 320 | G.41 | | | | 48h I | PSI-N; calmodulin binding [Arabidopsis thaliana] | 3.00E-043 |
| 193-G41-48I | EX982094 | 570 | G.41 | | | | 48h I | Photosystem II 10 kDa polypeptide, chloroplast precursor (PII10) | 2.00E-055 |
| 78.4-G41-48I | EX982095 | 243 | G.41 | | | | 48h I | Photosystem II 22 kDa protein [Arabidopsis thaliana] | 3.00E-026 |
| 207-G41-48I | EX982096 | 408 | G.41 | | | | 48h I | Light harvesting chlorophyll A/B binding protein [Prunus persica] | 4.00E-033 |
| **General metabolism** | |  |  | | | |  |  |  |
| 4.3-M.26-2I | EX982052 | 390 | M.26 | | | | 2h I | MYB11 [Malus x domestica] | 2.00E-067 |
| 1.2-M.26-2I | EX982056 | 320 | M.26 | | | | 2h I | Putative hydroquinone glucosyltransferase; arbutin synthase [Malus x domestica] | 9.00E-057 |
| 2.2-M.26-2I | EX982057 | 640 | M.26 | | | | 2h I | Glucose-6-phosphate dehydrogenase [Pisum sativum] | 1.00E-101 |
| 205-G41-48I | EY437146 | 434 | G.41 | | | | 48h I | Glyceraldehyde-3-phosphate dehydrogenase [Panax ginseng] | 7.00E-050 |
| 115-G41-2I | EX982122 | 380 | G.41 | | | | 2h I | Chalcone synthase [Malus x domestica] | 2.00E-064 |
| 98-G41-48I | EX982123 | 630 | G.41 | | | | 48h I | Putative chalcone isomerase 4 [Glycine max] | 3.00E-064 |
| M.26-2R | EX982053 | 209 | M.26 | | | | 2h R | Flavanone 3-hydroxylase [Malus x domestica] | 2.00E-019 |
| 171.2-G41-48I | EX982077 | 243 | G.41 | | | | 48h I | Beta-glucosidase [Malus x domestica] | 2.00E-023 |
| 92.2-G41-48I | EX982078 | 623 | G.41 | | | | 48h I | Malate dehydrogenase, NAD-dependent, eukaryotes and gamma proteobacteria [Medicago truncatula] | 4.00E-039 |
| 116-G41-2I | EX982079 | 412 | G.41 | | | | 2h I | Ripening-induced protein [Fragaria vesca] | 3.00E-036 |
| 210-G41-48I | EX982080 | 140 | G.41 | | | | 48h I | Ripening-related protein-like [Malus x domestica] | 2.00E-009 |
| 198.1-G41-48I | EX982081 | 125 | G.41 | | | | 48h I | Phospholipase [Malus x domestica] | 7.00E-024 |
| 90.1-M.26-48OE | EX982082 | 475 | M.26 | | | | 48h OE | Ubiquitin [Musa acuminata] | 2.00E-023 |
| M26-48I | EX982054 | 431 | M.26 | | | | 48h I | Polyubiquitin [Lilium longiflorum] | 2.00E-015 |
| 25.1-G41-48I | EX982083 | 479 | G.41 | | | | 48h I | putative desaturase-like protein [Trifolium repens] | 5.00E-048 |
| **Energy** |  |  |  | | | |  |  |  |
| 128-M.26-48I | EX982058 | 246 | M.26 | | | | 48h I | FED A; electron transporter/ iron ion binding [Arabidopsis thaliana] | 5.00E-029 |
| 139-G41-48I | EX982097 | 470 | G.41 | | | | 48h I | Iron superoxide dismutase [Arabidopsis thaliana] | 8.00E-011 |
| 191-G41-2I | EX982098 | 469 | G.41 | | | | 2h I | ATP binding / nucleoside diphosphate kinase [Arabidopsis thaliana] | 1.00E-021 |
| 214.1-G41-48I | EX982099 | 520 | G.41 | | | | 48h I | ATP binding / protein binding [Arabidopsis thaliana] | 1.00E-087 |
| 79.1-G41-48I | EX982116 | 335 | G.41 | 48h I | | | | Chloroplastic quinone-oxidoreductase homolog (ceQORH) | 6.00E-042 |
| 189-G41-48I | EX982121 | 460 | G.41 | | | | 48h I | NADP sorbitol-6-phosphate dehydrogenase [Malus x domestica] | 3.00E-084 |
| **Nucleic acid metabolism** | |  |  | | | |  |  |  |
| 214-G41-48I | EX982124 | 472 | G.41 | | | | 48h I | DnaJ protein homolog (DNAJ-1) emb|CAA47925.1| cs DnaJ-1 [Cucumis sativus] | 7.00E-075 |
| 55.1-M.26R | EX982059 | 310 | M.26 | | 2 and 48h I | | | Putative chromatin remodelling complex ATPase chain ISWI [Malus x domestica] | 3.00E-021 |
| 35.1-M26-48I | EX982060 | 126 | M.26 | | | | 48h I | RNA-binding region RNP-1 (RNA recognition motif) [Medicago truncatula] | 6.00E-004 |
| 54.2-M.26R | EX982061 | 240 | M.26 | | | 2 and 48h R | | DNA topoisomerase II [Malus x domestica] | 6.00E-025 |
| 55.2-M.26R | EX982062 | 255 | M.26 | | | 2 and 48h R | | SIR2-family protein [Malus x domestica] | 1.00E-043 |
| 114-G41-2I | EX982100 | 410 | G.41 | | | | 2h I | Deoxyribodipyrimidine photolyase, class 1 [Medicago truncatula] | 2.00E-016 |
| **Transport** |  |  |  | | | |  |  |  |
| 161-G41-48 | EX982101 | 350 | G.41 | | | | 48h I | zinc finger family protein [Fragaria x ananassa] | 2.00E-023 |
| **Unkown and unclassified** | |  |  | | | |  |  |  |
| 124.2-M26I | EX982070 | 291 | M.26 | | | 2 and 48h I | | ORF64c [Pinus koraiensis] | 4.00E-015 |
| 108-G41-48I | EX982102 | 350 | G.41 | | | | 48h I | F27F5.5 [Arabidopsis thaliana] | 2.00E-035 |
| 131.3-G41-48OE | EX982103 | 351 | G.41 | | | | 48h OE | Unknown [Arabidopsis thaliana] | 5.00E-017 |
| 170-G41-48I | EX982104 | 509 | G.41 | | | | 48h I | Unknown [Arabidopsis thaliana] | 2.00E-023 |
| 174-G41-48I | EX982105 | 520 | G.41 | | | | 48h I | Unknown [Medicago sativa] | 2.00E-023 |
| 176.1-G41-48I | EX982106 | 420 | G.41 | | | | 48h I | Unknown [Malus x domestica] | 2.00E-023 |
| 131.4-G41-48OE | EX982107 | 198 | G.41 | | | | 48h OE | Unknown |  |
| 66.2-G41-48R | EX982108 | 280 | G.41 | | | | 48h R | Unknow protein [Oryza sativa (japonica cultivar-group)] | 2.00E-039 |
| 72.1-M.26-2I | EX982063 | 282 | M.26 | | | | 2h I | Os01g0790900 [Oryza sativa (japonica cultivar-group)] | 7.00E-013 |
| 99.3-M26-2R | EX982064 | 262 | M.26 | | | | 2h R | Os01g0790900 [Oryza sativa (japonica cultivar-group)] | 2.00E-010 |
| 96-G41-48I | EX982110 | 340 | G.41 | | | | 48h I | Hypothetical protein OsJ_016228 [Oryza sativa (japonica cultivar-group)] | 2.00E-023 |
| 136.2-G41-2I | EX982111 | 723 | G.41 | | | | 2h I | Hypothetical protein 12.t00009 [Asparagus officinalis] | 0.011 |
| 48.1-G41-48OE | EX982113 | 291 | G.41 | | | | 48h OE | Hypothetical protein [Citrus x paradisi] | 9.00E-038 |
| 129.1-G41-48I | EX982114 | 291 | G.41 | | | | 48h I | Hypothetical protein [Citrus x paradisi] | 1.00E-037 |
| 127-G41-OE | EX982115 | 300 | G.41 | | 2 and 48h I | | | Hypothetical protein [Citrus x paradisi] | 5.00E-039 |
| 99.4-M26-2R | EX982065 | 540 | M.26 | | | | 2h R | Hypothetical protein, | 3.00E-017 |
| 84.2-M.26-2I | EX982066 | 276 | G.41 | | | | 2h I | Activated in Blocked Unfolded protein response family member (abu-10) [Caenorhabditis elegans] | 0.002 |
| 37-G41-48R | EX982117 | 138 | G.41 | | | | 48h R |  |  |
| 54-M.26R | EX982067 | 149 | M.26 | | 2 and 48h R | | |  |  |
| 126.2-M.26-48OE | EX982068 | 130 | M.26 | | | | 48h OE |  |  |
| 3.3-M.26-2I | EX982069 | 340 | M.26 | | | | 2h I |  |  |

^a^ time where the EST where found Induced (I), Repressed (R) or over-expressed in the AFLP analysis.
