# Supplementary material for: Identification of genes differentially expressed during interaction of resistant and susceptible apple cultivars (Malus × domestica) with Erwinia amylovora
Source: BMC Plant Biol. 2010 Jan 4;10:1. doi: 10.1186/1471-2229-10-1 (PMC2827420; doi:10.1186/1471-2229-10-1)
Supplement: Additional file 2 — DNA sequence of forward and reverse PCR primers used to confirm differential expression of specific ESTs. list of primer developed to study the expression of each specific EST which seems to be specifically activated or repressed during the interaction Malus Erwinia amylovora. [file 1471-2229-10-1-S2.DOC]

Table S2. DNA sequence of forward and reverse PCR primers used to confirm differential expression of specific ESTs.

|  | Primer sequence (5’ to 3’) |
| --- | --- |
|  |  |
| 4.2-M.26-2I ATP binding / kinase/ protein serine/threonine kinase | ttccgtccgatgagtttttc  ggcctctctttcgggttatc |
| 12-G41-48I putative aquaporin | GCGTATCGGTATGGAATGCT  CCATGACCAACTGACCACAG |
| 55.2-M.26R SIR2-family protein | gctgtagagcctggttgagc  ggggaaggaaaatccacagt |
| 84.2-M.26-2I unknown protein | CAAGCTGGAGTACGGTAGGG  CAGCGCCTAGTATCCATCGT |
| 98-G41-48I chalcone isomerase 4 | Gggtttacctggatgctgaa  caaggcttcctcctcctctt |
| 115-G41-2I chalcone synthase | caccaacagcgagcataaga  ggctgtccccattccttaat |
| 137.1-G41-48I unknown | ACAAGCTTTTAGGCCCCACT  AGGAGCACCACCTCTGCTAA |
| 137.2-G41-48I hypothetical protein B2 | GATCCAACTGCTTGGGAAGA  TAGCTCCAGGGTCTCAGGAA |
| 142-G41-48I Serine/threonine-protein kinase HT1 | aggtgatggagcagcagttt  tcgccaagaactgcctaact |
| 165-M.26-2R protein kinase | tcgtgtcaagattgctttgg  gctgtacccagatcctgcat |
| 166-M.26-2R protein kinase | ggttggatattgcatgactgg  gctagtcctttggcagatcc |
| 171.1-G41-48I protein kinase | ttgttcaaaagggcttacgg  tcctctccatcaccaacctc |
| 171-G41-48I putative leucine-rich repeat transmembrane protein kinase | ttctggcacatttcctctga  aagctgcagaaggggcttac |
| 175.2-G41-48I beclin 1 protein | Ttcgacttggacgacttcct  aagttcaccggaccaaacag |
| 175-G41-48I putative WRKY transcription factor 30 | Tagcacaagggagggatct  atgacctgtgggctgttgtt |
| 176.1-G41-48I unknown | ttcttcctgaatgggattgg  agctgcacatccctcaagtt |
| 176.2-G41-48I putative disease resistance protein | Atccaaagacttcgcaatgg  gctgaacaagctggaaaagg |
| 176.3-G41-48I protein kinase | Agggaagcttgttgatctcg  ctccaactcagcagtcagca |
| 177-G41-48I putative senescence-associated protein SAG102 | Tggttgcgtgagtgagagaa  ctcgactgcaaaaagccttc |
| 190-G41-48I Leucine-rich repeat | ggaatcccccattctcatct  cttgagaatccggagagcag |
| 194.5-G41-48I ELIP1 (early light inducible protein) | tagccaaccttccattccag  cagctccggagagaatcaac |
| 200.1-G41-48I Probable WRKY transcription factor 29 | ggcgtaagtatgggcaaaaa  tgtttcgacgagttggatga |
| 201-G41-48I translation initiation factor eIF-4A | Ctcatcccgtgttctcatca  caacacccttccttccaaat |
| 213-G41-48I Probable WRKY transcription factor 65 | Gtccaccaatttcagcgact  gcatccctttgagctactgc |
| 221-G41-48I WRKY-A1244 | gtccaccaatttcagcgact  gcatccctttgagctactgc |
| 200-G41-48I NSF attachment protein | TGAACAGCTGGGGATTTTTC  GCAATGTGCAGCATCAACA |
| 201.3-G41-48I LRR transmenbrane protein Kinase | gagataagacacaggaacattgtga  cctttttctccaccccactt |
| 201.M26R LYTB-like protein | AAGCTCAATCATGGCGAGTT  GATGATGCCCAACTTGGATT |
| 4.3-M.26-2I MYB11 | gcagggcttaagagatgtgg  cagaacatgagcgaccttca |
